# High-throughput screening for biostimulant discovery: opportunities to enhance nitrogen use efficiency

**DOI:** 10.3389/fpls.2026.1906676

**Published:** 2026-08-28

**Authors:** Mirthe Van Buyten, Dominique Audenaert, Tom Beeckman, Hans Motte

**Affiliations:** 1 Department of Plant Biotechnology and Bioinformatics, Ghent University, Ghent, Belgium; 2 Center for Plant Systems Biology, VIB, Ghent, Belgium; 3 VIB Screening Core, Ghent, Belgium

**Keywords:** biostimulants, discovery, nitrogen use efficiency (NUE), plant screening, screening assays

## Abstract

Improving plant nitrogen use efficiency (NUE) is essential for reducing fertilizer inputs, costs, and environmental impacts such as nitrate leaching and nitrous oxide emissions. Biostimulants that modulate nitrogen transporters, metabolic enzymes, or signaling regulators hold considerable promise in this regard. However, such biostimulants are largely discovered empirically and rarely act by specifically targeting these NUE-related processes. Despite major advances in structural and mechanistic knowledge in the plant nitrogen uptake, metabolism or signaling machinery, discovery screens that directly target these molecular components for the identification of NUE-enhancing biostimulants have, to our knowledge, rarely been reported. This contrasts with other areas of plant biology, where chemical genetics and high-throughput screening have yielded influential molecular tools and agrochemical leads. This review argues that high-throughput screening represents an untapped opportunity for biostimulant discovery aimed at improving the plant NUE. We outline key conceptual principles of such screening approaches and provide a comprehensive overview of the molecular networks governing nitrogen uptake, transport, assimilation, and signaling. By linking these molecular targets to tractable assay formats and screening strategies, we highlight concrete routes for the discovery of next-generation biostimulants, with a particular focus on small-molecule and other non-microbial products. Together, we aim to inspire the transition from empirical discovery toward mechanism-guided approaches to discover new biostimulants and open routes toward more sustainable, nitrogen-efficient crop production.

## Introduction

Plants are sessile organisms that have to cope with their environment during growth and reproductive stages. Under current climate change scenarios, more frequent and extreme weather events increasingly expose crops to abiotic stresses-such as heat, cold, drought, waterlogging, and salinity-which limit crop productivity and threaten global food security. Adequate nutrient availability is essential for both stress tolerance and optimal crop performance (Gong et al., 2020; Kumari et al., 2022). Therefore, modern agriculture relies heavily on fertilizers, particularly nitrogen, to sustain high crop yields and global food production (Xu et al., 2012; Fathi, 2022).

However, the production of nitrogen fertilizers has a high cost and energy demand. On top of this, agricultural crops typically take up only 30-50% of applied nitrogen fertilizers (West et al., 2014; You et al., 2023; Alami et al., 2025), leaving a large fraction wasted and contributing to environmental pollution. In soil, plant roots absorb nitrogen primarily as nitrate (NO_3_^-^) and ammonium (NH_4_^+^). Ammonium can be converted to nitrate by soil microorganisms, while nitrate easily leaches into the groundwater and aquatic ecosystems, contributing to eutrophication and biodiversity loss. Different soil microbial processes further convert unused nitrogen into gaseous nitrogen species, including the potent greenhouse gas nitrous oxide (N_2_O) (Beeckman et al., 2018, 2024).

Taken together, while fertilizer application is indispensable for maintaining crop productivity and supporting abiotic stress tolerance, a sustainable agricultural future requires strategies that allow stable yields while reducing nitrogen inputs and losses. This can be achieved by improving the plant nitrogen use efficiency (NUE), which is broadly defined as the efficiency with which a plant acquires nitrogen from its environment and converts it into biomass and yield. NUE is influenced by a range of processes, including nitrogen uptake, transport, assimilation, remobilization, and their signaling networks (Xu et al., 2012).

Over the past decade, plant nitrogen biology has advanced markedly. Mechanistic and structural work has clarified nitrate acquisition and signaling that tune root architecture and NUE (Wang et al., 2018b; Pélissier et al., 2021). These insights revealed molecular control points that may be modulated to improve NUE. Alongside genetic engineering, biostimulants represent a promising approach for influencing such processes (Quille et al., 2025). A biostimulant is a product stimulating plant nutrition processes independently of the product’s nutrient content, with the sole aim of improving nutrient use efficiency, tolerance to abiotic stress, quality traits, and/or availability of confined nutrients in the soil or rhizosphere (EU, 2019). Biostimulants offer a practical and flexible means to modulate plant biological processes, as they do not require genetic modification and can be applied across different crops and cultivars. However, a major challenge remains the identification of biostimulants that act on specific molecular pathways or traits directly related to NUE. Addressing this gap is essential for developing mechanism−guided solutions to improve nitrogen efficiency in a sustainable manner. In this review, we outline the principles of high−throughput screening approaches for biostimulant discovery, with a particular focus on non−microbial biostimulants that directly affect plant processes, including purified natural products and synthetic compounds, as high-throughput screening methodologies are most advanced for these product classes. Nevertheless, many of the concepts discussed could also be applicable to the discovery of other biostimulants that enhance NUE.

## Nature of biostimulants

Plant biostimulants have received increasing scientific and commercial attention over the past decades, driven largely by the need for more sustainable crop production systems and improved resource use efficiency (Calvo et al., 2014; Brown and Saa, 2015; Yakhin et al., 2017; Nephali et al., 2020; Ruzzi et al., 2024). Biostimulants are broadly defined as substances or microorganisms that, when applied to plants or the rhizosphere, stimulate physiological processes supporting improved growth, nutrient acquisition, and stress tolerance (du Jardin, 2015; EU, 2019) and are hence distinguished not by their composition, but by the nature of the response they induce. They are commonly grouped as microbial or non−microbial. Microbial biostimulants typically include plant growth−promoting rhizobacteria and arbuscular mycorrhizal fungi. Non−microbial biostimulants comprise humic and fulvic acids, protein hydrolysates, amino-acid-based products, macro- and micro-algae extracts, plant extracts, chitin and chitosan−based materials, as well as other complex organic mixtures derived from fermentation of animal or plant feedstock, food−processing by−products, and industrial streams (Calvo et al., 2014; du Jardin, 2015; Ruzzi et al., 2024). Furthermore, defined organic compounds, including small synthetic or natural molecules, represent an emerging class of biostimulants. Their high purity and known chemical structure enables a more precise elucidation of their mode-of-action, providing clearer insight into the biological processes they influence. In contrast, mechanistic understanding of complex materials such as seaweed extracts, humic substances, and plant hydrolysates, is significantly hampered by the presence of numerous bioactive constituents and potential interactions with the plant microbiome. While many products have demonstrated positive impacts on nutrient efficiency, stress resilience, and plant vigor, responses often remain species−, environment−, and dose−dependent, and batch−to−batch variability in natural materials can contribute to inconsistent performance in field condition (Brown and Saa, 2015; Yakhin et al., 2017; Nephali et al., 2020). Still, multiple biostimulants in the form of beneficial microorganisms or (derivatives of) natural substances have been shown to improve plant growth and to enhance NUE in plants, but their mechanism is often not known (Calvo et al., 2014; Brown and Saa, 2015; Yakhin et al., 2017; Nephali et al., 2020; Ruzzi et al., 2024). For example, treatment with the PSI-362 fraction from the seaweed *Ascophyllum nodosum* extracts, results in an increased nitrate accumulation in Arabidopsis and in increased NUE in barley grains (Goñi et al., 2021). Likewise, treatment with the amino acid-based biostimulants Terra Sorb^®^ radicular or Terramin^®^ Pro results in an increased biomass, enhanced photosynthesis, accumulation of nitrate/total nitrogen and improved NUE (Navarro‐León et al., 2022).

Historically, most biostimulants have been discovered through low-throughput, empirical, and often serendipitous approaches. This trial-and-error mode of discovery has yielded important products, but it inherently limits the pace and precision of identifying defined, mechanistically tractable bioactive agents. In contrast, recent developments in chemical genetics and high-throughput screening offer a powerful alternative to discover NUE-enhancing biostimulants.

## High-throughput screening

### Screening libraries as a source for new biostimulants

High-throughput screening enables rapid and systematic evaluation of thousands or more products. Today, a wide range of commercial and academic screening libraries containing tens of thousands products are readily available, and form a valuable resource for candidate biostimulants.

Many of these libraries are accessible in formats compatible with plant-focused high-throughput screening, such as pre-plated 96- or 384-well configurations. A large set of libraries contain pure individual molecules, typically selected to maximize chemical diversity, while meeting empirically defined physicochemical criteria that promote solubility and biological uptake. Many collections have been optimized for pharmaceutical discovery (Warr et al., 2022), but plant-oriented libraries also exist. For example, the Agro−like library from Enamine is enriched for scaffolds prevalent in known agrochemicals while filtering out toxic or non−agrochemical motifs (https://enamine.net/compound-libraries/targeted-libraries/agro-like-library). In addition, diverse natural product or natural product-like libraries are available (e.g. https://www.medchemexpress.com/screening/Natural_Product_Library_.html; https://www.targetmol.com/compound-library/Natural_Product_Library). Although these natural collections tend to be smaller - typically a few thousand molecules - they provide chemotypes with inherent biological relevance and generally greater public acceptance for agricultural applications. In this respect, also large libraries of crude or prefractionated natural−product extracts can be easily acquired (https://www.nccih.nih.gov/grants/natural-product-libraries), and significantly expand the accessible chemical diversity of natural products (Thornburg et al., 2018). Beyond the use of the crude or fractionated extracts as a possible biostimulant, such extracts can also serve as starting material for the identification of the natural active products through iterative round of activity-guided subfractionation (Li et al., 2024).

Next to diversity oriented libraries, many targeted libraries are available that contain focused sets around certain scaffolds, molecules with known activity toward certain biological processes or well-annotated molecules. One such resource relevant for plant biology is the Library of AcTive Compounds in Arabidopsis (LATCA), a carefully curated set of small molecules demonstrating bioactivity in Arabidopsis (Chow, 2007) and applied in multiple Arabidopsis chemical screening efforts (e.g. Zhao et al., 2007; Schreiber et al., 2008; Abdel-Hamid et al., 2011; Drakakaki et al., 2011; Stokes et al., 2013; Bonnot et al., 2016; Carland et al., 2016; Fiers et al., 2017; Sakai et al., 2017). Bioactive or annotated libraries assembled from compounds with known targets or pathways in other organisms (including FDA−approved drugs or clinical candidates) may facilitate repurposing and accelerate mode-of-action elucidation in plants (Dejonghe and Russinova, 2017).

More recently, AI−enabled custom libraries started to emerge, in which generative models and predictive machine−learning tools are used to mine enormous virtual chemical spaces and assemble focused sets of molecules tailored to specific biological targets (Segler et al., 2018). For example, structure-based virtual pre-screening strongly increases the efficiency of hit discovery compared with untargeted screening collections (Gentile et al., 2022; Zhou et al., 2024).

Taken together, these traditional and next-generation libraries create unprecedented opportunities to identify novel biostimulants, including those enhancing NUE. However, realizing this potential depends on robust plant screening assays.

### Plant screening strategies

To achieve a high scale, high-throughput screening relies on miniaturized and standardized assay formats, typically implemented in multi-well plates, allowing large compound libraries to be tested in parallel. This requirement imposes important constraints on assay design, as assays are limited in physical scale, while readouts must be preferentially rapid, quantifiable, and compatible with automated imaging or detection systems. As a result, such screens often favor simplified or proxy readouts, such as fluorescent or enzymatic reporters, rather than complex morphological or physiological phenotypes (Audenaert and Overvoorde, 2014; Serrano et al., 2015).

Overall, two types of screening approaches can be distinguished: forward or phenotypic screening versus reverse or target-based screening. In forward or phenotypic screening, compounds are selected based on a biological output, without prior knowledge of the molecular target. Classical plant screens often rely on morphology or reporter expression, but can also use a wide variety of other biological responses like protein localization, signaling dynamics, metabolite levels, cellular physiology, etc (Serrano et al., 2015; Dejonghe and Russinova, 2017). Typical assays use seedlings or cell cultures expressing a fluorescent reporter (such as GREEN FLUORESCENT PROTEIN (GFP)) or an enzymatic reporter (such as β-glucuronidase (GUS)) under control of a promoter responsive to the process of interest. After treatment with a compound library, hits are identified as those causing an altered biological response.

In contrast, reverse, or target−based, screening starts with a known protein or complex, and compounds are selected based on their ability to modulate that target, typically using biochemical or biophysical assays that report on its activity or conformational state. Although this approach is standard in pharmaceutical discovery, target-based screening is rather uncommon in plant science (Dejonghe and Russinova, 2017). A main constraint is the need of functional proteins for such assays, while producing functional plant proteins is often difficult. Moreover, compound-induced responses heavily depend on uptake and metabolism, which is absent in *in vitro* assays.

A high-throughput compound screen typically consists of a primary screen to select candidate compounds in a primary assay, followed by a confirmation screen to verify their effects and to eliminate false positives. In the primary screen, often only one biological repeat per compound is used as current libraries contain a large number of compounds (10.000-100.000 compounds or more). Running even a second replicate would double reagent consumption, plate usage, equipment occupation time, data storage requirements, and overall cost. The primary screen primarily aims to maximize throughput to filter a huge number of candidates down to a manageable subset. Although this inevitably risk false positives, these will be removed during the confirmation screen, which often includes multiple repeats per compound. Moreover, primary assays are ideally optimized to be highly robust and sensitive, making one measurement per compound sufficient to detect possible effects. Therefore, and to optimize the cost-efficiency, primary assays are in general miniaturized into 96-well or 384-well plate formats and have ideally an automated readout. Next to false positives, false negatives are an inherent risk in high-throughput screening, as any assay may fail to identify compounds with genuine biological activity. Such false negatives may arise from limitations of the assay design itself, but also because biostimulant responses are often dose-dependent, while compounds in a primary screen are typically tested at a single concentration. Consequently, compounds tested outside their optimal concentration range may be overlooked during the primary screen, while hit compounds should be further subjected to dose-response analyses in follow-up phases (see below).

High-throughput screening in whole plants has been pioneered primarily in the model plant *Arabidopsis thaliana*. Here, seedlings are typically grown and treated in 96-well plates in liquid medium (Audenaert et al., 2014; Nguyen et al., 2018; Huang and Li, 2021), although other formats, using for example solid medium or even soil are possible as well (De Diego et al., 2017; Li et al., 2023). Preferably, more than one seedling is grown per well to improve assay robustness and buffer biological variation. Depending on the biological question, these assays can use wild-type seedlings or specific mutants. Micro- or macroscopic phenotyping can be used to monitor changes in growth, morphology or development (e.g. De Rybel et al., 2009; De Diego et al., 2017; Li et al., 2023). Reporter lines can be used to provide more targeted and sensitive readouts, enabling microscopic imaging or biochemical assays and monitoring of transcriptional activity, protein localization, signal transduction, etc (e.g. Surpin et al., 2005; De Rybel et al., 2012; Kerchev et al., 2014; Meesters et al., 2014; Xiong et al., 2014; Halder and Kombrink, 2015; Fiers et al., 2017). Commonly used reporters include GUS for biochemical detection or GUS staining, luciferase for quantitative luminescence-based assays or fluorescent reporters enabling microscopic assessment. Initially, most plant compound screens relied on macroscopic phenotypes or GUS staining. However, the qualitative nature of GUS assays complicates consistent quantification. In contrast, luciferase reporters, although less suitable for subcellular or tissue-contextual analyses due to their limited spatial resolution, offer a fully quantitative alternative (Halder and Kombrink, 2015; Fiers et al., 2017). Fluorescence-based plant screens offer high-resolution spatial information, but typically require substantial manual assessment. Yet with the emerging automated and AI−assisted image analysis pipelines, fluorescence−based assays are increasingly becoming fully quantifiable and scalable (Jiang, 2024).

Although whole plants remain the most physiologically relevant system, the use of intact seedlings inherently limits assay miniaturization and throughput, and complicates subcellular and cellular imaging or biochemical analyses. Moreover, it is difficult to use crop species in whole-plant assays. Organ−level assays alleviate many of these constraints. Excised leaves, for example, enable uniform compound exposure and easier optical access, making them amongst others suitable for high-resolution fluorescence imaging, ratiometric biosensor readouts and luminescence imaging (Boruc et al., 2010; Kitawaki et al., 2023). Likewise, other organs such as root explants, stem segments, or coleoptile tip sections enable the study of specific developmental or physiological contexts while allowing more controlled imaging (e.g. Nishimura et al., 2012, 2014; Motte et al., 2013; Verstraeten et al., 2013). On the other hand, distribution and manipulations of such organs are often labor-intensive and difficult to automize.

Assay possibilities and throughput can be further increased by the use of cell-based systems, which are compatible with 384- or even 1536-well formats. Cell−based assays include both cell suspension cultures and protoplasts. Suspension cultures are stable, continuously maintained populations of dividing cells, suitable for highly reproducible assays and suitable for high-throughput screening. Protoplasts, which are cells from which the cell wall is removed by an enzymatic treatment, must be freshly prepared for each experiment, but offer substantially greater flexibility. They can be generated from a wide range of species, including crop species such as maize, rice, and wheat and can be transiently transfected. Cell-based assays can be used for various reporter analyses and biochemical assays (Noutoshi and Shirasu, 2018; Gilliard et al., 2021; Chen et al., 2023; Reyna-Llorens et al., 2023) and are compatible with both plate-reader quantification (e.g. De Vriese et al., 2019) and high-throughput or high-content imaging (e.g. Yoneda et al., 2007; Drakakaki et al., 2011; Yang et al., 2024).

### Targeting plant nitrogen use efficiency

**Figure 1:**
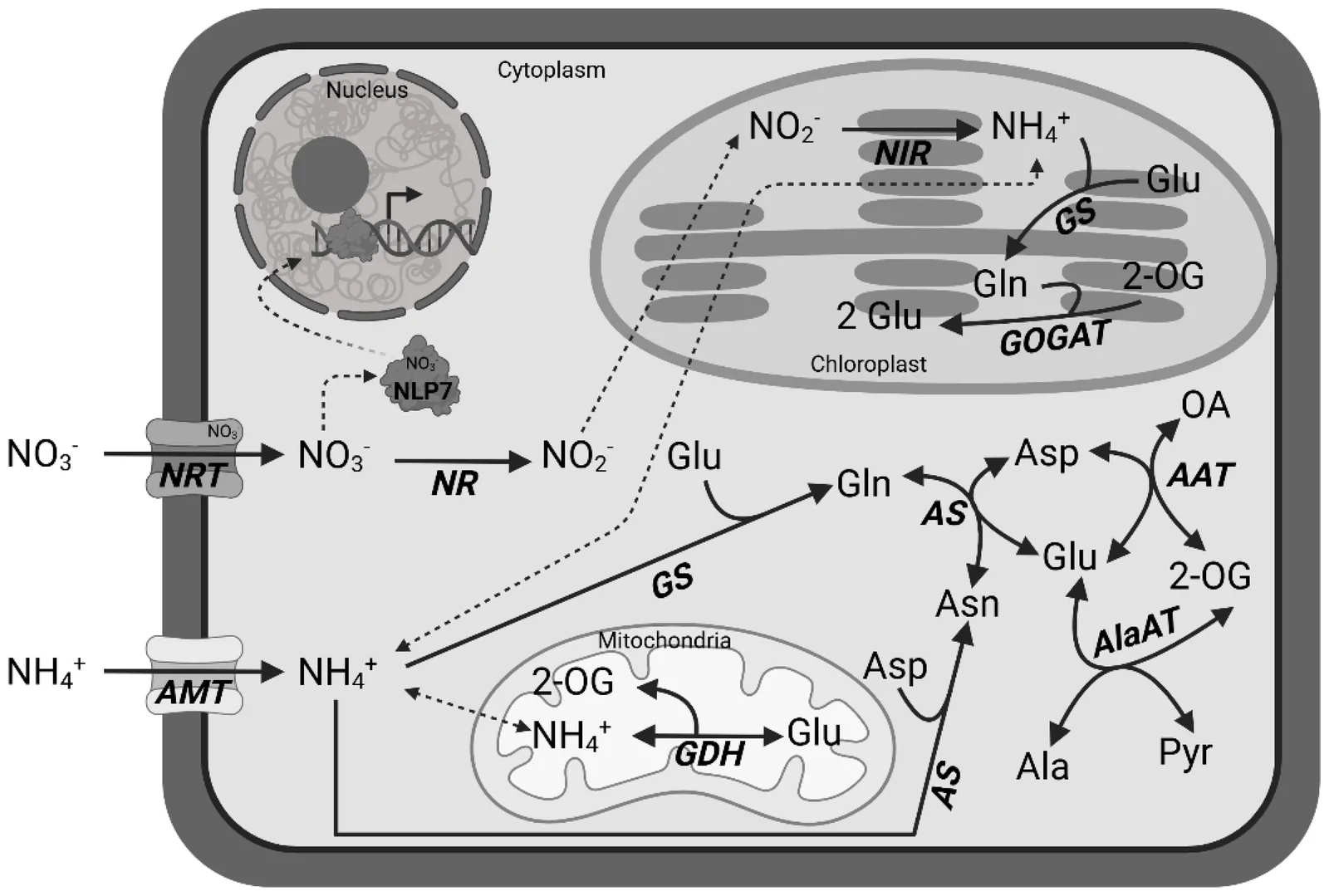
Selection of important molecular players and molecules in nitrogen transport and nitrogen assimilation in plants. NRT, NITRATE TRANSPORTER; AMT, AMMONIUM TRANSPORTER; NLP7, NODULE INCEPTION-LIKE PROTEIN 7; NR, NITRATE REDUCTASE; NIR, NITRITE REDUCTASE; GS, GLUTAMINE SYNTHETASE; GOGAT, glutamine-2-oxoglutarate aminotransferase; GDH, GLUTAMATE DEHYDROGENASE; AS, asparagine synthetase; AAT, aspartate aminotransferase; AlaAT, ALANINE AMINOTRANSFERASE; NO_3_^-^, nitrate; NH_4_^+^, ammonium; NO_2_^-^, nitrite; Glu, glutamate; Gln, glutamine; 2-OG, 2-oxoglutarate; Asp, aspartate; Asn, asparagine; OA, oxaloacetate; Ala, alanine; Pyr, pyruvate. Image made in BioRender (https://app.biorender.com/illustrations/684adcd4f243df4d3b8b9563).

Targeted NUE-improving biostimulant discovery strategies requires a clear identification of the molecular components underlying plant NUE, which is largely determined by two processes: nitrogen uptake/transport and nitrogen assimilation (**Figure 1**) (Xu et al., 2012; Li et al., 2017). Further on, during reproductive development, nitrogen remobilization through source-to-sink transport of amino acids from senescing vegetative tissues to developing seeds, grains, or fruits can contribute substantially to NUE and final crop productivity (Tegeder and Masclaux-Daubresse, 2018).

The main nitrogen forms, ammonium and nitrate, are taken up by the root cells via AMMONIUM TRANSPORTER (AMT) or NITRATE TRANSPORTER (NRT) proteins, respectively. The major contributor to nitrate uptake is NRT1.1, which is a dual affinity nitrate transporter that facilitates uptake under both low or high external nitrate concentrations. Other low-affinity nitrate uptake transporters (e.g. NRT1.2) or high-affinity NRT2/NAR2 complexes are specifically responsible for uptake under high or low nitrate conditions, respectively (Tsay et al., 2007; Laugier et al., 2012; McDonald et al., 2022; Xu et al., 2024). NRT2 proteins are dephosphorylated in presence of nitrate, which results in structural flexibility enabling nitrate transport (Xu et al., 2024). In contrast, constitutive low affinity nitrate transport occurs by passive entry of nitrate through large hydrophilic loop between transmembrane domains of the epidermal NRT1.2 protein (Tsay et al., 2007). The dual affinity transport of nitrate by NRT1.1 is regulated by phosphorylation of threonine 101 (Thr101) and dimer (de)coupling: when nitrate concentrations are high, Thr101 is not phosphorylated and NRT1.1 remains in a dimeric state, which structurally supports the low−affinity transport. Moreover, in such conditions, nitrate binds both NRT1.1 monomers, stabilizing the dimer. When nitrate levels are low, nitrate will only bind on one NRT1.1 monomer (the high-affinity monomer) which will trigger Ca^2+^ waves that are sensed by the calcium sensor calcineurin B-like protein 9 (CBL9) which activates the CBL-interacting protein kinase 23 (CIPK23) to phosphorylate Thr101. This causes dimer decoupling into a monomeric state, resulting in increased structural flexibility which ensures high affinity transport (Liu et al., 1999; Liu and Tsay, 2003; Sun et al., 2014; Rashid et al., 2020; Fang et al., 2021; McDonald et al., 2022).

Ammonium is mainly taken up by the high-affinity ammonium transporters AMT1;1 and AMT1;3, which forms homotrimers or heterotrimers in the plasma membrane with a central pore for ammonium conduction (Yuan et al., 2013). When ammonium levels are high, the conductivity of this pore will be inhibited by C-terminal phosphorylation, while the transporters will become degraded via clathrin-mediated endocytosis, reducing uptake to prevent toxicity that is caused by high ammonium levels (Neuhäuser et al., 2007; Loqué et al., 2009; Wang et al., 2013; Hao et al., 2020).

After ammonium and nitrate uptake, radial transport towards the stele occurs through a combination of apoplastic movement in the inter-cellular space and cell-to-cell symplastic movement. At the endodermis, the radial apoplastic flow is stopped by casparian strips, and nitrate and ammonium enter the symplast mainly via NRT1.1 or NRT2.1/NAR2.1 and AMT1;2, respectively (Nazoa et al., 2003; Neuhäuser et al., 2007; Léran et al., 2013; Duan et al., 2018; Bao et al., 2019). AMT2.1 and NRT1.5 exports ammonium and nitrate from the pericycle cells into the xylem for their root-to-shoot translocation (Lin et al., 2008; Dechorgnat et al., 2011; Giehl et al., 2017). NRT1.8 has an opposite role, and removes nitrate from the xylem for redistribution in case of stress conditions (Li et al., 2010), while NRT1.9 limits root-to-shoot translocation by facilitating nitrate transport in the opposite direction under high nitrate availability (Wang and Tsay, 2011).

Of note, nitrate assimilation into amino acids occurs in most plants, in particular herbaceous non-legumes, primarily in the shoot (Andrews and Raven, 2022). There, other NRT proteins unload nitrate from the xylem in the leaves (e.g. NRT1.4) or to the phloem (e.g. NRT1.11 and NRT1.12) for sink delivery (e.g., seeds, young leaves) (Chiu et al., 2004; Hsu and Tsay, 2013; Krapp et al., 2014; Tegeder and Masclaux-Daubresse, 2018). In addition, NRT1.7, expressed in older source leaves, mediates phloem loading of nitrate for redistribution towards younger sink tissues (Fan et al., 2009). Ammonium, in contrast to nitrate, is primarily assimilated in roots and is generally less redistributed over long distances (Hachiya and Sakakibara, 2017).

In the leaves, the enzyme NITRATE REDUCTASE (NR) reduces nitrate to nitrite (NO_2_^-^) in the cytoplasm. Nitrite is further reduced by NITRITE REDUCTASE (NIR) into ammonium, typically in chloroplasts (**Figure 1**). In both roots and shoots, ammonium is incorporated into amino acid metabolism through three main enzymatic routes. The primary and energetically favored pathway is the GS–GOGAT cycle: GLUTAMINE SYNTHETASE (GS), located in the cytosol and plastids, combines ammonium and glutamate to form glutamine, after which the glutamate synthase glutamine-2-oxoglutarate aminotransferase (GOGAT) transfers the amide group to 2−oxoglutarate to regenerate two molecules of glutamate. A second pathway involves asparagine synthetase (AS) in the cytosol, which incorporates ammonium indirectly by converting aspartate and glutamine into asparagine and glutamate (Kissen et al., 2010; Xu et al., 2012). Third, GLUTAMATE DEHYDROGENASE (GDH), a mitochondrial and plastidial enzyme, is able to catalyze the reversible reaction between ammonium and 2-oxoglutarate to form glutamate. Of note, GDH functions mainly in glutamate deamination and nitrogen recycling rather than primary ammonium assimilation (Kissen et al., 2010; Xu et al., 2012; Dellero, 2020; Wang et al., 2024b), and there is, to our knowledge, no convincing genetic evidence linking GDH manipulation to improved NUE.

Following ammonium assimilation into glutamate and glutamine, aminotransferases transfer the amino group of glutamate onto a wide range of carbon skeletons to produce other amino acids (Kissen et al., 2010; Xu et al., 2012; Dellero, 2020; Koper et al., 2025) (**Figure 1**). For example, aspartate aminotransferase (AAT) catalyzes the reversible transfer of this amino group to oxaloacetate, generating aspartate, the precursor for several essential the amino acids including lysine, threonine and methionine (Kissen et al., 2010). Likewise, ALANINE AMINOTRANSFERASE (AlaAT), transfers the amino group of glutamate to pyruvate to form alanine and 2−oxoglutarate, thereby linking nitrogen metabolism tightly to glycolysis and carbon flux (Kissen et al., 2010; McAllister and Good, 2015; Dellero, 2020; Tiong et al., 2021). The branched-chain aminotransferase (BCAT) is important as well and essential for the final biosynthetic step of leucine, valine, and isoleucine (Xing and Last, 2017). Because aminotransferase reactions rely on carbon skeletons such as oxaloacetate, pyruvate, and 2−oxoglutarate, amino acid biosynthesis is a highly energy-demanding process and places substantial demands on the tricarboxylic acid (TCA) cycle activity, mitochondrial respiration, and photosynthetic carbon assimilation (Foyer et al., 2011; Xu et al., 2012; Pratelli and Pilot, 2014; Hildebrandt et al., 2015; Zhang and Fernie, 2023). Consequently, stimulation of nitrogen assimilation and aminotransferase activity is typically coupled to the upregulation of carbon metabolism to sustain the required reducing capacity and carbon skeletons.

After vegetative growth, developing seeds acquire nitrogen mainly as amino acids from degrading proteins in senescing leaves, and to a lesser extent from post-flowering nitrogen uptake that is assimilated directly in the seeds, or in leaves or other vegetative organs before being exported to the seeds (Xu et al., 2012; Tegeder and Masclaux-Daubresse, 2018). These amino acids, mainly glutamine and asparagine, are transported via the phloem towards the seeds and are unloaded in the seed by USUALLY MULTIPLE ACIDS MOVE IN AND OUT TRANSPORTER (UMAMIT) proteins and by AAP amino acid permeases. There, glutamine and asparagine undergo deamidation by glutaminase and asparaginase to release ammonium, which is then reassimilated via the GS-GOGAT cycle to fuel subsequent amino acid biosynthetic pathways via different aminotransferases. Although members of both the AAP and UMAMIT transporter families contribute to source-to-sink nitrogen transport, genetic evidence linking improved NUE to transporter manipulation is currently stronger for *AAP* (**Table 1**) than for *UMAMIT* genes (Besnard et al., 2021).

**Table 1:** Examples of genetic approaches targeting molecular players involved in nitrogen uptake, transport or assimilation, or their controlling factors, that are shown to improve NUE or yield.

Category	Gene/homologue	Function	Genetic modification	Reported phenotype(s)	Validated crops	Reference(s)
Nitrate transport	NRT2.1	High-affinity nitrate uptake	Overexpression/nitrate-inducible expression (NAR2.1 promotor)	Increased nitrate uptake and NUE	Rice, oilseed rape	(Chen et al., 2016; Naz et al., 2019; Nan et al., 2024)
Nitrate transport	NRT2.3	High-affinity nitrate uptake	Overexpression	Increased nitrate uptake, grain yield and NUE	Rice	(Fan et al., 2016)
Nitrate transport	NAR2.1	Essential partner protein for NRT2 transporters	Overexpression	Increased nitrate uptake, yield and NUE	Rice	(Chen et al., 2020)
Nitrate transport	NAR2.1 + NRT2.3	High-affinity nitrate uptake module	Overexpression	Increased nitrate uptake, yield and NUE	Rice	(Chen et al., 2020)
Nitrate transport	NRT1/OsNPF7.6/OsNPF8.20/LeNRT2.3	Low-affinity nitrate transport	Overexpression	Increased nitrate uptake, grain/fruit yield and NUE	Rice, tomato	(Fang et al., 2013; Fu et al., 2015; Zhang et al., 2022)
Nitrate transport/signaling	NRT1.1	Dual-affinity nitrate transporter and receptor	Overexpression/introgression of natural variant with stronger promoters	Improved nitrate uptake efficiency under low N	Maize	(Allen et al., 2016; Sakuraba et al., 2021)
Nitrate transport/signaling	NRT1.1/OsNRT1.1B	Dual-affinity nitrate transporter and receptor	Introgression of allele with modified phosphorylation/signaling capacity	Improved nitrate uptake efficiency, grain yield and NUE	Rice	(Hu et al., 2015)
Nitrate transport/signaling	NRT1.1/OsNRT1.1A	Dual-affinity nitrate transporter and receptor	Overexpression	Improved NUE and yield, shortened maturation time	Rice, wheat	(Wang et al., 2018a, 2024a)
Nitrate signaling	NLP7/OsNLP4	Transcription factor, master regulator of nitrate-induced gene expression	Overexpression	Increased yield and NUE	Rice	(Wu et al., 2021b)
Nitrate signaling	NLP7/OsNLP4	Transcription factor, master regulator of nitrate-induced gene expression	Introgression of allele with enhanced NRE binding capacity.	Increased yield and NUE	Rice	(Wu et al., 2025)
Nitrate signaling	CBL9/OsCBL1	Ca^2+^ sensor regulating CIPK activity toward NRT1.1	Knock-down	Increased nitrogen accumulation and NUE at low nitrogen levels	Rice	(Hu et al., 2023)
Ammonium transport	AMT1;1	Ammonium uptake	Overexpression	Potential increased ammonium uptake, NUE and grain yield (studies also report growth penalties or toxicity at moderate to high ammonium levels)	Rice	(Hoque et al., 2006; Kumar et al., 2006; Ranathunge et al., 2014)
Ammonium transport	AMT1;2	Ammonium root-to-shoot transport	Overexpression combined with Glutamate synthetase activation	Increased tolerance to nitrogen limitation, improved ammonium uptake and nitrogen remobilization	Rice	(Lee et al., 2020a)
Amino acid transport	AAP1	Amino acid import into seeds	Seed cotyledon and/or phloem-specific overexpression	Increased seed nitrogen and potentially increased seed size and number	Pea, Narbon bean	(Rolletschek et al., 2005; Weigelt et al., 2008; Zhang et al., 2015; Perchlik and Tegeder, 2017)
Assimilation	NiR	Nitrite reduction	Nitrate-induced expression	Enhanced nitrogen assimilation, more tillers, higher yield & NUE	Rice	(Yu et al., 2021)
Assimilation	GS1	Glutamine synthesis	Overexpression/addition of extra gene copy	Improved NUE, nitrogen content, biomass and grain yield	Rice, maize, wheat, barley	(Habash et al., 2001; Oliveira et al., 2002; Ortega et al., 2004; Martin et al., 2006; Cai et al., 2009; Brauer et al., 2011; Gao et al., 2019; Wu et al., 2021a)
Assimilation	GOGAT	Glutamate synthesis	Overexpression	Improved NUE, grain yield and biomass in tobacco and rice, but decreased growth in maize (presumably due to imbalance between nitrogen and carbon)	Rice	(Chichkova et al., 2001; Yamaya et al., 2002; Cañas et al., 2020)
Assimilation	AlaAT	Aminotransferase	(Root-specific) Overexpression	Improved NUE, biomass and seed yield	Rice, wheat, barley, sorghum, oilseed rape	(Good et al., 2007; Shrawat et al., 2008; Peña et al., 2017; Selvaraj et al., 2017; Sisharmini et al., 2019; Tiong et al., 2021)
Assimilation	AS	Asparagine synthesis	Overexpression	Increased grain nitrogen content	Rice	(Lee et al., 2020b)
Nitrogen uptake (by deep rooting)	DRO1	Regulation of root gravitropic response	Introgression of a natural variant	Increased nitrogen uptake and grain yield	Rice	(Arai-Sanoh et al., 2014)

Most of the molecular players involved in the nitrogen uptake or transport, or the transcription of the genes encoding them, are activated by the presence of ammonium and/or nitrate (Pélissier et al., 2024; Shanks et al., 2024). Although ammonium uptake and assimilation pathways are fairly well characterized, the upstream mechanisms inducing ammonium-dependent responses remain poorly characterized. In contrast, nitrate signaling is better understood. More specifically, NRT1.1 proteins do not only transport nitrate, but also function as primary nitrate sensors, acting as bona fide nitrate receptors that initiate downstream signaling cascades (Ho et al., 2009). Upon nitrate perception, NRT1.1 rapidly triggers changes in cytosolic Ca^2+^ concentrations to activate Ca^2+^−dependent protein kinases (CPKs). These CPKs subsequently phosphorylate nitrate−responsive transcription factors, including NODULE INCEPTION-LIKE PROTEIN (NLP7), mediating its nuclear retention and leading to the binding on promotors with nitrate response elements (NREs), and as such directly controlling hundreds of genes, including many genes encoding players involved in nitrogen transport and assimilation (Marchive et al., 2013; Liu et al., 2017; Varala et al., 2018; Alvarez et al., 2020). Moreover, NLP7 is a nitrate receptor as well: binding of nitrate triggers a conformational change that enables its activity as a transcription factor (Liu et al., 2022).

Beyond direct modulation of nitrogen transport, signaling, or assimilation pathways, biostimulants may also improve NUE indirectly through effects on root system architecture, thereby increasing the root uptake area and enhancing nitrogen foraging. Several nitrate-responsive regulators, including NRT1.1 and its downstream signaling network, contribute to both root system architecture and NUE, illustrating the close connection between root development and nitrogen acquisition (Pélissier et al., 2021). In contrast to screens directly targeting NUE-associated molecular players, multiple screens targeting root-affecting factors are reported (e.g. Armstrong et al., 2004; He et al., 2011; De Rybel et al., 2012). These studies generally focused on hormonal or root developmental pathways, while the potential impact of the identified compounds on NUE was not investigated. Yet, an important caveat is that the relationship between root system architecture and NUE is highly context-dependent, and different root architectures may be favorable under different nitrogen conditions (Lynch, 2019; Motte et al., 2019). This complicates the identification of root architectural traits or molecular players that can be broadly targeted by biostimulants to improve NUE. Nevertheless, some root ideotypes, such as the steep and deep rooting phenotype, which can be stimulated by affecting gravitropic response genes such as *DEEPER ROOTING1* (*DRO1*), *ALTERED RESPONSE TO GRAVITY1* (*ARG1*) or *ENHANCED GRAVITROPISM2* (*EGT2*) (Boonsirichai et al., 2003; Uga et al., 2013; Kirschner et al., 2021), have shown promise for improving nitrogen acquisition by facilitating the capture of mobile nitrate from deeper soil layers (Lynch, 2013, 2019; Arai-Sanoh et al., 2014).

### From molecular targets to screening assays

**Figure 2:**
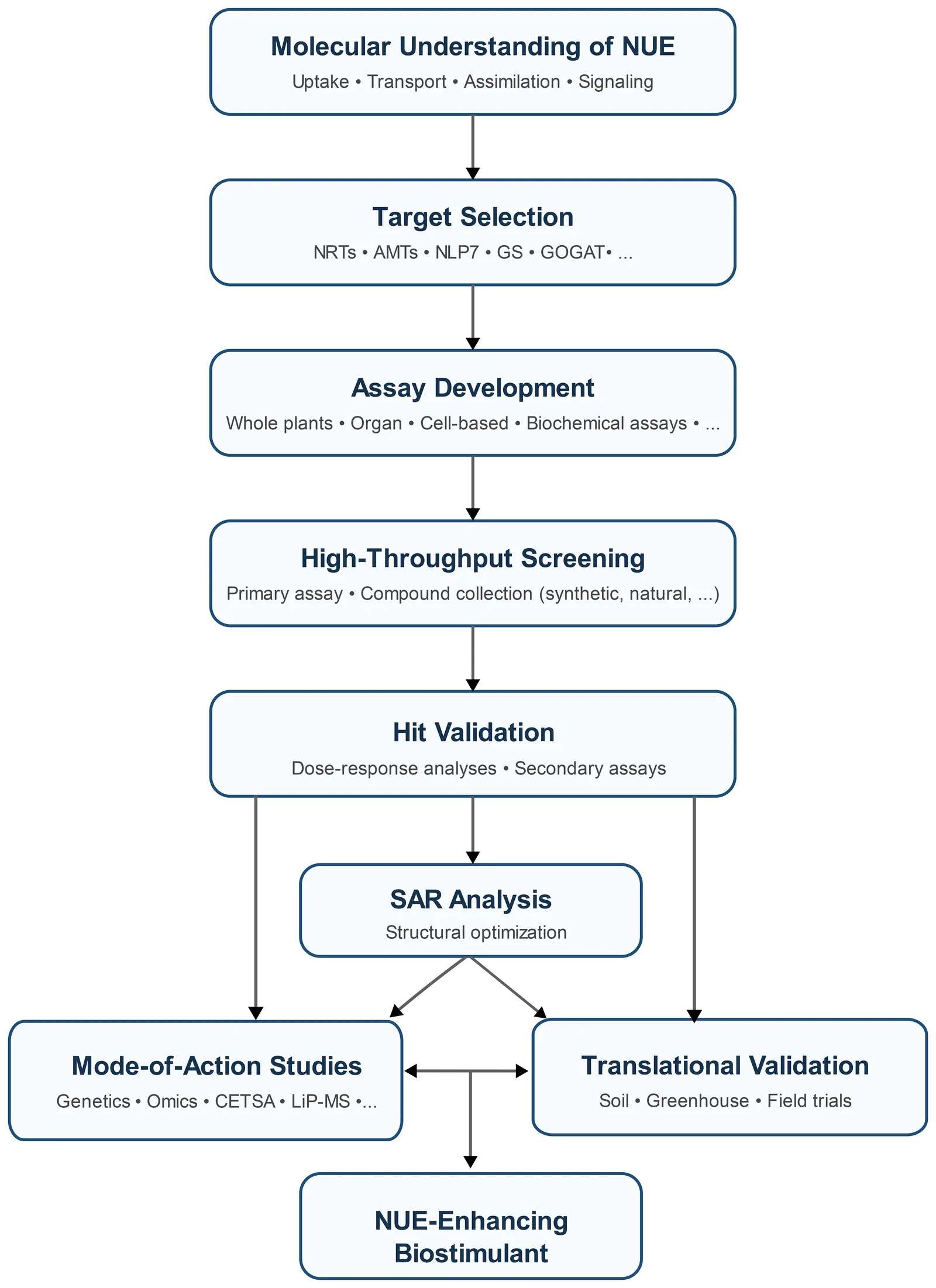
Workflow for high-throughput screening and discovery of NUE-enhancing biostimulants. Current knowledge of the molecular mechanisms underlying nitrogen uptake, transport, assimilation, and signaling provides candidate targets for screening. These targets can be translated into reporter-, cell-, or biochemical-based assays suitable for high-throughput screening of compound libraries using a primary assay. Candidate hits are subsequently validated through dose-response analyses and secondary assays, while optimized via structure-activity relationship (SAR) analyses. Further characterization via mode-of-action studies can support mechanistic insights useful for the regulatory process and for product credibility. Finally, candidate biostimulants should be evaluated in increasingly complex environments, progressing from soil-based assays and greenhouse experiments to field trials, to assess their robustness and agronomic relevance.

The increasing understanding of the molecular mechanisms underlying nitrogen uptake, transport, assimilation, and signaling enables the development of discovery pipelines for NUE-enhancing biostimulants (**Figure 2**). Molecular players involved in these processes represent promising targets to enhance NUE, although their increased activity may also affect other processes or lead to metabolic imbalances and hence cause growth penalties or a decreased NUE. Still, genetic approaches targeting several of these genes and modifying their expression or activity have demonstrated positive effects on NUE or yield under certain conditions (**Table 1**), supporting their potential contribution to NUE. Hence, biochemical or transcriptional assays that monitor the expression or activation state of these genes offer a potential mean to identify candidate biostimulants capable of enhancing NUE.

In practice, several assay formats could be deployed to screen for compounds that activate these pathways. For example, Arabidopsis reporter lines carrying promoter::luciferase, GUS or GFP fusions of key nitrate−responsive genes could be used to detect compounds that induce the same transcriptional programs known to improve NUE. Similarly, reporters based on the promoters of the transport or assimilation genes would allow detection of biostimulant candidates that induce those genes. In addition to transcriptional reporters, translational fusions with fluorescent reporters could report on the nuclear localization, for example of NLP proteins in protoplasts (Castaings et al., 2009; Liu et al., 2017; Hu et al., 2019). Fluorescent split biosensors could indicate conformational changes that increase transcription activity of NLP7 (Liu et al., 2022).

Together, such readouts provide concrete, mechanistically grounded strategies to identify compounds capable of stimulating the same targets, processes, or signaling nodes that have proven effective through genetic modification, thereby accelerating the discovery of next−generation biostimulants that enhance NUE.

## The post-screening process

Any hit in high-throughput screening assays designed to select possible biostimulants are only preliminary candidates, as the primary assay in principle only provides a simplified or partial proxy of plant performance in a miniaturized format and as such cannot capture the full physiological, developmental, or environmental complexity. Accordingly, such hits require a rigorous validation process to confirm and characterize their biological activity in a more relevant biological context. The exact validation steps depend on the biological question, the design of the primary assay and the availability of more complex secondary assays. In most workflows, validation begins with dose-response analyses in the primary assay to verify the effect and, where possible, optimize dosing, as the effect of biostimulants is typically dose-dependent. It is equally important to validate the effect in independent secondary assays that assess the same biological activity via alternative readouts. Because a compound may influence multiple processes beyond the selected readout, validation should also include broader phenotypic assessment to evaluate developmental and physiological consequences of the candidate biostimulants. Depending on the intended biostimulant application, validation in soil-based systems will be critical, as plant NUE is strongly influenced by rhizosphere processes, microbial activity, and nitrogen cycling in the soil. Moreover, compounds may undergo microbial degradation or transformation. As a result, biostimulant efficacy can differ substantially from that observed in simplified laboratory assays. A step−wise progression from controlled lab−scale tests, to greenhouse experiments, and, where appropriate, field trials, is essential to demonstrate translatability under increasingly realistic conditions and assess agronomic relevance (Li et al., 2024). In addition, the progression from laboratory hit to commercial product may be influenced by formulation requirements and regulatory considerations, which can differ between synthetic small molecules, purified natural compounds, and more complex biostimulant products. While these considerations are important for successful product development, a detailed discussion of these aspects is beyond the scope of the present review.

During initial validation steps, it is also of interest to identify which structural features of the hit are essential for activity. This is typically achieved through a structure-activity relationship (SAR) analysis, in which a series of close analogues, derivatives, or fragment variants of the original hit are systematically tested (Tóth and Van Der Hoorn, 2010). This may reveal compounds with increased potency, higher specificity, lower complexity, or other properties relevant for commercial production or formulation. It also aids in deducing which substructures of the molecule could be altered without loss of activity. These modifiable regions are particularly valuable for downstream mode-of-action studies as they can be substituted with affinity tags, photo-reactive groups, or clickable handles to enable target identification approaches such as affinity-purification mass spectrometry (AP-MS).

This brings us to one of the most challenging phases in biostimulant discovery: the identification of the target(s) of the compound and deciphering its mode-of-action (MOA). Although not absolutely required, such MOA information is particularly valuable for application, as it improves product credibility, provides insights into potential off-target or undesirable effects. Moreover, MOA studies can help validate that the observed NUE effect results from the modulation of specific plant processes, thereby providing mechanistic justification to support regulatory acceptance.

The choice and order of experimental techniques to elucidate the MOA will depend strongly on the research question and on the nature of the phenotype observed. In practice, MOA discovery typically requires an integrated strategy combining multiple experimental approaches, ranging from genetics and biochemistry to imaging, and omics-based profiling, and integrating them with existing pathway knowledge (Overvoorde and Audenaert, 2013; Xuan et al., 2013).

Careful characterization of phenotypic or cell-biological responses can indicate the affected pathway. If the compound induces a recognizable and scorable phenotype, classical genetic approaches are often the most straightforward entry point. Screening for mutant lines that are insensitive or hypersensitive to the compound can reveal genes required for its biological effect. Although such genes do not necessarily encode the direct target, they often highlight pathways that are important for the MOA of the compound,.

Omics profiling after compound treatment is another powerful approach for generating MOA hypotheses. Multiple omics approaches, including transcriptomics, (phospho-/chemo-)proteomics and metabolomics, can provide complementary evidence about relevant genes, pathways and signaling modules influenced by the compound. Transcriptome changes often capture early signaling responses and can infer pathway similarity or gene-expression modules by comparison to reference datasets. Metabolomics adds functional depth by detecting changes in metabolic fluxes and biochemical pathway activity, which is particularly relevant when compounds act on nutrient assimilation or stress-responsive metabolic circuits (Shen et al., 2023). Proteomic and phosphoproteomic shifts provide a closer view of signaling dynamics and post−translational regulation. A particular useful approach for direct target identification is to perform proteomics after affinity capture of compound−interacting proteins, as this delivers direct candidate binders. This is achieved by adding a specific tag to the compound and immobilizing it on a column. Then, its interacting proteins are captured from an extracted protein mixture using affinity purification and identified using mass spectrometry (MS). The chemical tagging is not always possible, as it may affect the activity of the compound, and hence prevent binding to its relevant target. Label-free assays are an alternative approach that can be used to identify proteins that undergo compound-induced structural or stability changes. Typical label-free assays include assays based on thermal denaturation, such as cellular thermal shift assay (CETSA) or thermal proteome profiling (TPP) (Lu et al., 2022; Ma et al., 2022; Tolvanen, 2022; Lu and Russinova, 2023), or on protease accessibility, such as limited proteolysis mass spectrometry (LiP−MS) (Venegas-Molina et al., 2023; Chen et al., 2026).

These approaches may offer valuable entry points to candidate targets of the biostimulant, but more detailed investigation is required to validate these targets. In case the target protein contains a specific enzymatic activity, biochemical assays using purified or enriched proteins offer a direct functional approach to assess whether the compound modulates the activity of the potential target enzyme. Biophysical binding assays, including Microscale Thermophoresis (MST) and Surface Plasmon Resonance (SPR), are valuable methods to provide evidence that the compound is able to physically interact with the target protein (Ziegler et al., 2013; Huang and Zhang, 2021). Just as the biochemical assays, these techniques require purified or highly enriched protein. However, purifying plant proteins is often challenging, as these are often embedded in membranes or protein complexes. Alternatively, rescue-based approaches can be used to position a compound within known biological pathways by probing whether its induced phenotype can be mitigated, reversed, or modified. Such rescue effects can be achieved through multiple strategies, including the exogenous supply of metabolites or pathway intermediates, genetic modifications (e.g. knockout or overexpression lines), or pharmacological treatments using known inhibitors or activators.

When a compound-induced phenotype is alleviated by one of these interventions, it can provide important clues about the level at which the compound acts within a metabolic or signaling cascade. In addition, combinatorial treatments that result in antagonistic or synergistic effects can further refine pathway positioning and reveal functional relationships between biological processes. Although these approaches do not necessarily identify the direct molecular target, they provide a powerful framework to localize compound activity within biological networks and to generate testable hypotheses about its MOA. In this way, they form an essential complementation to biochemical and biophysical methods in the iterative process of MOA elucidation.

## Conclusions and perspectives

Biostimulants, including beneficial microorganisms, natural compounds, and synthetic molecules, hold significant potential to enhance plant NUE in agricultural systems. While most currently available biostimulants have been identified empirically, advances in our understanding of the molecular mechanisms governing nitrogen uptake, transport, assimilation, and signaling now provide a strong foundation for more targeted discovery strategies.

A key opportunity lies in the development of high−throughput screening platforms that translate this molecular knowledge into tractable and scalable assays. Designing such screens requires careful consideration of assay format and readout, favoring robust, quantitative, and miniaturized systems compatible with high-throughput formats. These may include phenotypic assays based on simplified proxies of NUE, or target-oriented approaches that monitor the activity or regulation of defined components of the nitrogen network, such as transporter function or pathway-specific gene expression.

Importantly, candidate compounds emerging from these screens require rigorous downstream validation. This includes dose–response characterization, confirmation across complementary assays, and evaluation under increasingly realistic conditions, progressing from controlled environments to soil-based and field systems. In parallel, structure-activity relationship (SAR) analyses and mode-of-action (MOA) studies are essential to refine compound properties, identify molecular targets or affected pathways, and establish mechanistic understanding.

Together, the integration of high−throughput discovery, mechanistic validation, and translational testing provides a coherent framework to move from molecular knowledge to practical applications. By adopting such approaches, future research can transition from largely empirical biostimulant discovery toward a more rational and predictive paradigm, ultimately enabling the development of next-generation solutions for sustainable and nitrogen-efficient crop production.
